# Arabidopsis NITRILASE 1 Contributes to the Regulation of Root Growth and Development through Modulation of Auxin Biosynthesis in Seedlings

**DOI:** 10.3389/fpls.2017.00036

**Published:** 2017-01-24

**Authors:** Thomas Lehmann, Tim Janowitz, Beatriz Sánchez-Parra, Marta-Marina Pérez Alonso, Inga Trompetter, Markus Piotrowski, Stephan Pollmann

**Affiliations:** ^1^Lehrstuhl für PflanzenphysiologieRuhr-Universität Bochum, Bochum, Germany; ^2^Centro de Biotecnología y Genómica de PlantasUniversidad Politécnica de Madrid (UPM)-Instituto Nacional de Investigación y Tecnología Agraria y Alimentación (INIA), Pozuelo de Alarcón, Spain

**Keywords:** *Arabidopsis thaliana*, auxin biosynthesis, nitrilases, indole-3-acetic acid, root development, GC-MS/MS

## Abstract

Nitrilases consist of a group of enzymes that catalyze the hydrolysis of organic cyanides. They are found ubiquitously distributed in the plant kingdom. Plant nitrilases are mainly involved in the detoxification of ß-cyanoalanine, a side-product of ethylene biosynthesis. In the model plant *Arabidopsis thaliana* a second group of *Brassicaceae*-specific nitrilases (NIT1-3) has been found. This so-called NIT1-subfamily has been associated with the conversion of indole-3-acetonitrile (IAN) into the major plant growth hormone, indole-3-acetic acid (IAA). However, apart of reported functions in defense responses to pathogens and in responses to sulfur depletion, conclusive insight into the general physiological function of the NIT-subfamily nitrilases remains elusive. In this report, we test both the contribution of the indole-3-acetaldoxime (IAOx) pathway to general auxin biosynthesis and the influence of altered nitrilase expression on plant development. Apart of a comprehensive transcriptomics approach to explore the role of the IAOx route in auxin formation, we took a genetic approach to disclose the function of NITRILASE 1 (NIT1) of *A. thaliana*. We show that NIT1 over-expression (NIT1ox) results in seedlings with shorter primary roots, and an increased number of lateral roots. In addition, NIT1ox plants exhibit drastic changes of both free IAA and IAN levels, which are suggested to be the reason for the observed phenotype. On the other hand, *NIT2*RNAi knockdown lines, capable of suppressing the expression of all members of the NIT1-subfamily, were generated and characterized to substantiate the above-mentioned findings. Our results demonstrate for the first time that Arabidopsis NIT1 has profound effects on root morphogenesis in early seedling development.

## Introduction

Auxins are major plant growth regulators that control virtually all aspects of plant growth and development, including embryo development, cell expansion growth, cambial activity, and formation of lateral and adventitious roots. Moreover, auxins also contribute to photo- and gravitropic responses, fruit ripening and senescence (Thimann, 1977; Davies, 2010). Despite the century-long history of auxin research, the elucidation of molecular mechanisms that control cellular auxin homeostasis and the activity of the pathways that contribute to auxin metabolism are still an active field of research. Nitrilases were among the first enzymes that were linked with the biosynthesis of indole-3-acetic acid (IAA), the most important auxin occurring in plant. Nitrilase activities have early been described from a broad variety of plant families including *Cruciferae, Gramineae*, and *Musaceae* (Mahadevan and Thimann, 1964; Thimann and Mahadevan, 1964). In particular after the identification of nitrilases in the model plant *Arabidopsis thaliana* they were suggested to be key enzymes of IAA biosynthesis in that they catalyze the conversion of indole-3-acetonitrile (IAN) to IAA (Bartling et al., 1992, 1994; Bartel and Fink, 1994). Obviously, this initial assumption did not stand the proof of time, since we know today that other enzymes, such as the members of the YUCCA-family, are the main players in auxin biosynthesis (Stepanova et al., 2011; Won et al., 2011).

**Figure 1:**
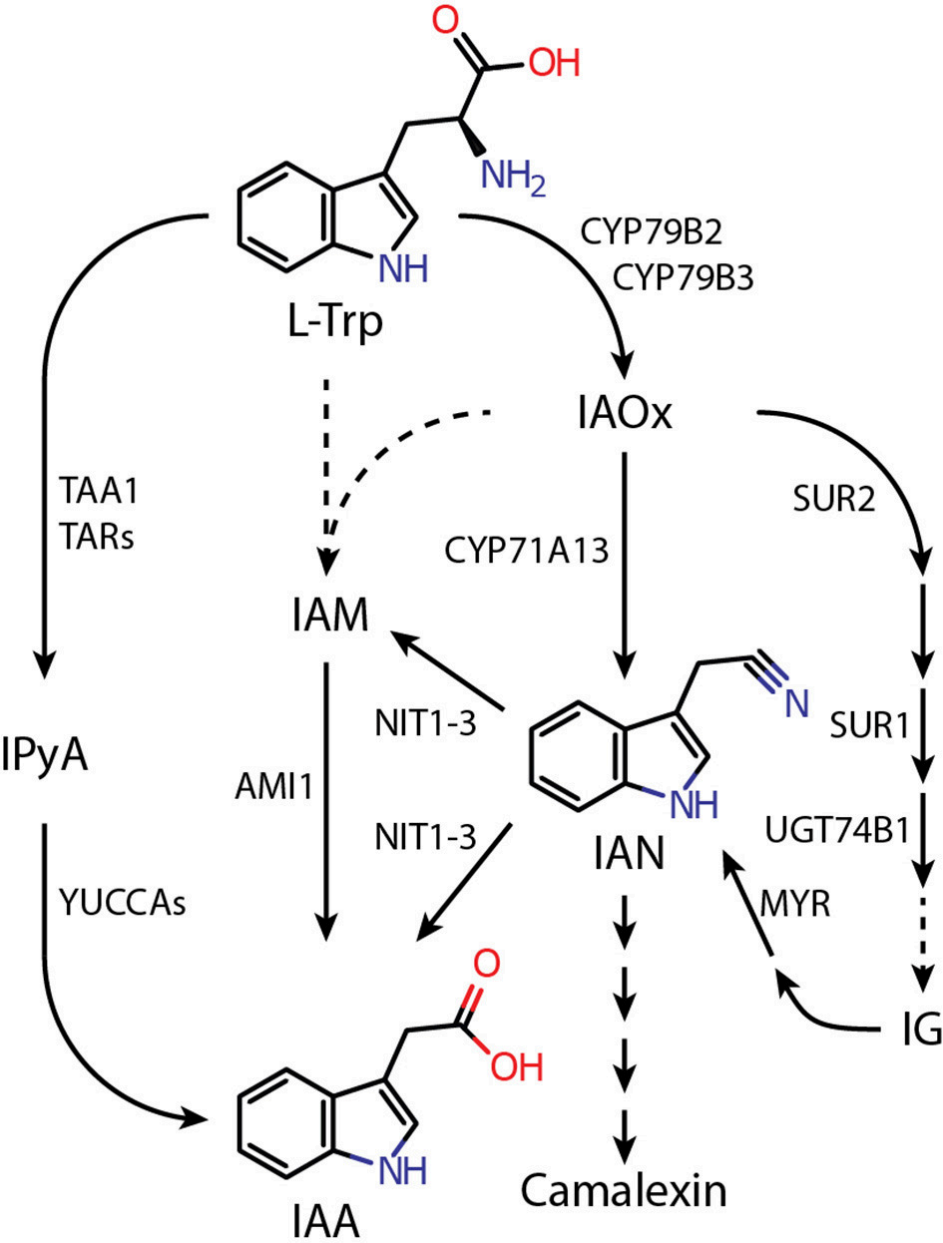
**Proposed pathways of L-tryptophan-dependent IAA biosynthesis in ***Arabidopsis thaliana*****. Dashed lines indicate assumed reaction steps for which the corresponding enzymes have yet to be identified. Metabolites and enzymes are abbreviated as follows: AMI1, AMIDASE 1; CYP71A13, CYTOCHROME P450 MONOOXYGENASE 71A13; CYP79B2/B3, CYTOCHROME P450 MONOOXYGENASE 79B2/B3; IAA, indole-3-acetic acid; IAM, indole-3-acetamide; IAN, indole-3-acetonitrile; IAOx, indole-3-acetaldoxime; IG, indole glucosinolate; IPyA, indole-3-pyruvic acid; L-Trp, L-tryptophan; MYR, MYROSINASE; NIT1-3, NITRILASE 1-3; SUR1, SUPERROOT 1 (S-ALKYL-THIOHYDROXIMATE LYASE); SUR2, SUPERROT 2 (CYP83B1); TAA1, TRYPTOPHAN AMINOTRANSFERASE OF ARABIDOPSIS 1; TAR, TRYPTOPHAN AMINOTRANSFERASE RELATED; UGT74B1, UDP-GLUCOSYL TRANSFERASE 74B1.

According to the current perception, auxin formation in plants involves several possible pathways that either act in parallel or in a redundant manner (Pollmann et al., 2006; Zhao, 2014; Kasahara, 2015). Arabidopsis, as a member of the *Brassicaceae*, possesses an additional pathway that proceeds through the intermediate indole-3-acetaldoxime (IAOx) (Figure 1). IAOx is considered to be an important metabolic branching point from which a number of different pathways emerge that can eventually lead to the production of either IAA or defense-related secondary metabolites, such as camalexin or indole glucosionlates (IGs) (Zhao et al., 2002; Glawischnig et al., 2004; Sugawara et al., 2009; Sønderby et al., 2010). However, the contribution of the IAOx pathway to general IAA biosynthesis is not yet clear. A number of studies have shown that a double mutant, *cyp79b2*/*cyp79b3*, compromised in the initial step of the IAOx pathway has wild type-like IAA levels under normal growth conditions but a modest decrease of free IAA levels when grown at 26°C (Zhao et al., 2002; Sugawara et al., 2009), suggesting a more substantial role for the IAOx pathway in IAA formation. Thus, far, it cannot be excluded that other pathways can efficiently compensate perturbations in IAA biosynthesis caused by genetic blockade in *cyp79b2*/*cyp79b3* under normal growth conditions.

The mutant plants *sur1* and *sur2*, which are characterized by a reduced flux of IAOx into IGs and increased IAA contents, display rather lower levels of IAN than wild-type Arabidopsis (Barlier et al., 2000; Sugawara et al., 2009). Moreover, the *sur2* phenotype is not suppressed in the *nit1* genetic background, which argues against parallel pathways and IAN being a direct metabolite of IAOx (Bak et al., 2001). However, the direct conversion of IAOx into IAN has been reported to mark the first step in camalexin biosynthesis and a role of IAN as an intermediate in this pathway has been confirmed. This reaction is catalyzed by the cytochrome P450 monooxygenase 71A13 (Nafisi et al., 2007). Expression of camalexin biosynthesis-associated genes is strongly induced by pathogens at the infection site but remains low under normal conditions (Glawischnig, 2007). Hence, it appears highly unlikely that CYP71A13 plays an important role in general IAN production. Based on these observations the most probable source for free IAN in Arabidopsis is the IG glucobrassicin. Glucobrassicin can be metabolized to IAN, and a number of publications reported a positive correlation between the levels of those two compounds (Müller and Weiler, 2000; Reintanz et al., 2001; Zhao et al., 2002).

The Arabidopsis genome contains four nitrilase isogenes, designated NIT1–NIT4. They fall into two clades according to the different functions of their gene products. Whereas the NIT4-subfamily only contains the phylogenetically oldest isoenzyme, NIT4, which has been shown to be involved in hydrogen cyanide detoxification (Piotrowski et al., 2001; Piotrowski and Volmer, 2006), the NIT1-subfamily consists of the isoenzymes NIT1–NIT3. The members of the NIT1-subfamily, which are thought to be restricted to the *Brassicaceae* (Janowitz et al., 2009), are suggested to have emerged from NIT4 by gene duplication events and subsequent neo-functionalization (Piotrowski, 2008). By this, the enzymes are assumed to have acquired new functions that, in turn, have helped to provide an evolutionary advantage to *Brassicaceae* plants.

Over the past years a couple of groups studied the function of Arabidopsis nitrilases in remarkable detail. Besides NIT4, also the members of the NIT1-subfamily could be associated with potential physiological functions. While NIT1 and NIT2 are assumed to be involved in the protection against bacterial pathogens (Grsic-Rausch et al., 2000), and NIT2 furthermore in terms of leaf senescence (Quirino et al., 1999), NIT3 seems to participate in the initiation of lateral root growth when the plants sustain sulfur deprivation (Kutz et al., 2002). In addition, a NIT1 deficient EMS mutant, *nit1-3*, has been described by Normanly et al. (1997), which showed an enlarged tolerance toward exogenously applied IAN while showing wild type sensitivity to IAA in the growth medium. Moreover, the authors were able to show that complementation of *nit1-3* by a 35S promoter driven *NIT2* expression was possible. This, in fact, corroborated the findings of Schmidt et al. (1996) that emphasized the general capability of Arabidopsis NIT2 to convert exogenously given IAN to IAA when expressed in tobacco.

Although several lines of evidence point out that IAN is converted to IAA in root tissue of Arabidopsis (Müller et al., 1998) and that nitrilase expression is induced by high levels of cellular IAN (Müller and Weiler, 2000), the work of Vorwerk et al. (2001) casted doubts on the involvement of nitrilases in the basal production of IAA, since *in vitro*-experiments revealed that IAN, the assumed direct IAA precursor, is not among the preferred substrates of the enzymes. This aspect is further underlined by the matter of fact that the complementation of *nit1-3* by over-expressing either *NIT1, NIT3*, or *NIT4* failed, although strong transcription of all 35S-driven *NIT* constructs has been confirmed by northern blot analysis (Normanly et al., 1997). However, overexpression of NIT1–NIT4 in these lines has not been studied on the post-translational level. Additionally, impairment of the *NIT1* gene was described to have neither a detectable effect on the steady state level of free IAA nor on the total IAA level (free + conjugated IAA) of the plant. Thus, evidence for a clear functional and enzymatic assignment for NIT1 has still to be supplied.

Here, we report on a whole-genome microarray approach to study perturbations in auxin biosynthesis through the genetic knockout of the IAOx pathway. In addition, we describe the construction and characterization of an Arabidopsis gain-of-function mutant, NIT1ox, a plant with obvious alterations in root morphogenesis. The mutant plants are distinguished by approximately 40% shorter primary roots, while showing an increased number of shorter lateral roots and root hairs, hence, pointing toward an over-production of the major plant growth hormone, IAA. Although the expression of NIT1 is not restricted to root tissue, no other plant organ showed a divergent phenotype. The phenotypic observations have been substantiated by an increased nitrilase activity in crude extracts of NIT1ox seedlings as well as by mass spectrometric analysis, revealing clear alterations of IAN and free IAA levels, which seem to be responsible for the detected developmental phenotype. Together, our findings suggest that changes in the cellular NIT1 content indirectly or directly affect auxin metabolism and root morphogenesis. In addition, we constructed and analyzed *NIT2*RNAi lines that are characterized by substantially reduced NIT1-3 contents. In particular one line, *NIT2* RNAi-8, displayed both immunologically not detectable NIT1-NIT3 levels and considerably decreased total IAA content, which confirms our previously described findings.

## Materials and methods

### Plant material and plant growth conditions

The experiments were carried out using *Arabidopsis thaliana* (L.) Heynh. ecotype Col-0 (originally from Nottingham Arabidopsis Stock Centre, NASC, stock N1092). *nit1-3* seeds were provided by the Nottingham Arabidopsis Stock Centre (NASC stock N3738) and seeds for the *cyp79b2*/*cyp79b3* double mutant was kindly provided by Dr. Hiroyushi Kasahara (RIKEN Center for Sustainable Resource Science, Yokohama, Japan). Seedlings were raised under sterile conditions on solidified ½-strength MS-medium supplemented with 1% (w/v) sucrose in Petri dishes (Murashige and Skoog, 1962); plantlets were kept under constant environmental conditions (8 h light at 24°C, 16 h darkness at 20°C, photosynthetically active radiation 105 μmol photons m^−2^ s^−1^ from standard white fluorescent tubes) for 2–3 weeks. If older plant material was used, the plant organs were harvested from plants grown in a greenhouse on a mixture of soil and sand (2:1) for 4–6 weeks in short days (8 h photoperiod). Thereafter, plants were transferred to long day conditions (16 h photoperiod). The greenhouse was maintained under constant climatic conditions, 22–24°C during daytime and 18–20°C over night. The photosynthetically active radiation was no less than 150 μmol photons m^−2^ s^−1^ (supplementary light, if required, from sodium-vapor lamps).

### Microarray analysis

Genome-wide expression studies with ATH1 arrays (Affymetrix) were performed using 2-week-old *cyp79b2*/*cyp79b3* and Col-0 plants, grown in chambers under 21/18°C, 16/8 h photoperiod. Three biological replicates were harvested and frozen into liquid N_2_ prior to RNA extraction. Total RNA was extracted following Oñate-Sánchez and Vicente-Carbajosa (2008). Arrays for the different plant materials were hybridized according the Affymetrix GeneChip Expression Analysis manual (http://www.affymetrix.com). Variation between replicates was accounted for by using the LIMMA model, which allows correlation of expression levels within each biological replicate and where information is drawn across genes by fixing the within-biological clone correlation (Smyth, 2005). Differentially expressed genes were identified by the modified *t*-test implemented in the LIMMA package. The LIMMA package uses a Benjamini-Hochberg correction to adjust for multiple testing. A *p*-value of 0.05 after adjustment for multiple testing and *log*2 ratio ≥1.2 were arbitrarily chosen to select 620 differentially expressed genes in *cyp79b2*/*cyp79b3* relative to wild-type Arabidopsis (Supplemental Data Sheet 1), which roughly corresponds to 2% of the genes. A more classical adjusted *p*-value of 0.005 maintaining a *log*2 ratio of ≥1.2 would select 462 genes. Of the 620 differentially regulated genes a total number of 491 genes could be assigned to GO ontologies and used for BiNGO analysis (Supplemental Data Sheet 2) using a hypergeometric test with a traditional Benjamini-Hochberg FDR correction and a post-correction selection significance level of 0.05 (Maere et al., 2005).

### Construction and validation of vectors for plant transformation

To fuse the full-length NIT1 protein with a C-terminal c-myc epitope-tag, the *NIT1* cDNA was amplified without its stop codon by PCR, with PM81 DNA as the template (Bartling et al., 1992), adding additional restriction sites for *Spe*I and *Not*I by using the following primer set: 5′-TATACTAGTATGTCTAGTACTAAAGAT-3′, 5′-ATATGCGGCCGCTTTGTTTGAGTCATCCTCA-3′. The PCR product was sub-cloned into pBluescript SK(+) and sequence integrity was verified by commercial sequencing (Qiagen, Hilden, Germany). Thereafter, the *Spe*I/*Not*I fragment was fused to the coding sequence of the c-myc epitope followed by a stop codon and a nos terminator region, resulting in the vector pBs-NIT1-cmyc. The prepared NIT1:c-myc fusion was subsequently transferred into the *Sma*I site of the expression vector pEXP1 (Raymond et al., 1990). To do so, pBs-NIT1-cmyc was sequentially digested with *Spe*I and *Sac*I, after each digestion the resulting fragment end was modified by either a complete Klenow fill-in (*Spe*I site) or an exonuclease reaction (*Sac*I site) using T4 DNA polymerase. After identifying a clone harboring the NIT1:c-myc fusion in the correct orientation (pEXP1-nit1), this clone served as a preliminary control to check the enzymatic activity of the NIT1:c-myc fusion protein prior to plant transformation. In case of the construct to induce RNAi in *A. thaliana*, a two-step cloning procedure was applied. At first, a fragment consisting of the second exon and intron of *NIT2* was amplified by PCR using a primer set that introduced restriction sites for *Xba*I/*Eco*RI: 5′-TATTCTAGAAAAAGGCGAACAAGTTTATTGTGG-3′, 5′-TATGAATTCCTACAGGTTCATCACACAAAAAAAG-3′. Secondly, only the second exon of *NIT2* was amplified, this time adding *Sac*I/*Eco*RI restriction sites: 5′-TATGAGCTCAAAAGGCGAACAAGTTTATTGTGGAG-3′, 5′-TATGAATTCCAGGAACTTTAATAGCAGAAGCATG-3′. Both fragments were cloned into the pGEM-T vector (Promega, Madison, USA) and sequence integrity was verified by sequencing (MWG Biotech, Ebersberg, Germany). The second exon was excised from pGEM-T using the introduced *Sac*I site and a *Pst*I site from the vector. The excised fragment was cloned into pBluescript SK(+) resulting in the Vector pBSNIT2E2. The fragment containing exon and intron two was cloned into the *Eco*RI/*Apa*I sites of pBSNIT2E2 giving rise to pBSNIT2RNAi containing the full RNAi construct. To construct binary vectors the *Spe*I/*Sac*I fragment from pBs-NIT1-cmyc and the *Xba*I/*Sac*I fragment from pBSNIT2RNAi, respectively, were introduced downstream of the cauliflower mosaic virus 35S promoter into the plant transformation vector pCBi18 (Biesgen and Weiler, 1999), replacing the originally included uidA gene.

### Heterologous expression of recombinant NIT1:c-myc

To investigate the general feasibility of NIT1:c-myc fusion constructs, the fusion protein was at first expressed in *Escherichia coli*, strain XL1-blue, and, thereafter, the nitrilase activity of the crude extracts was compared. To carry out these experiments, 100 mL cultures of 2xYT medium were freshly inoculated (1:25) with appropriate over-night cultures, containing bacteria harboring either the empty vector pEXP1 or the plasmid pEXP1-nit1, and incubated at 37°C under constant shaking and selection pressure (Amp, 100 μg ml^−1^) until an OD_600_ ≥ 0.6 was reached. Afterwards, adding 0.2 mM IPTG induced the protein expression. After 4 h, bacteria were harvested by centrifugation (5000 g, 15 min, 4°C) and pellets were re-suspended in 1/10 of the culture volume of lysis buffer (100 mM potassium phosphate buffer pH 8). Hereafter, complete cell disruption was achieved by six burst of ultrasound (20 s). The soluble protein fraction as well as the pellet fraction was obtained by centrifugation (6000 g, 15 min, 4°C). After separation of the supernatant and the pellet, the sediment was re-suspended in 1/10 of the culture volume of lysis buffer (*vide supra*).

### Generation of transgenic plants

Vectors carrying either the *35S::NIT1:c-myc* or the *35S::NIT2RNAi* fragment were introduced into the *Agrobacterium tumefaciens* strain C58C1 [pMP90] (Koncz and Schell, 1986) by electroporation. Incorporation of the binary vectors was verified by restriction analysis and polymerase chain reaction, using primers specific for the recombinant construct. Transgenic plants were generated following the protocol of Clough and Bent (1998). Seeds of transformed T_0_-plants were harvested in bulk, sown on soil, and selected by spraying with 0.025% (v/v) BASTA (Hoechst, Frankfurt, Germany), equivalent to 50 mg L^−1^ phosphinotricin (PPT), every second to third day (De Block et al., 1987). For *35S::NIT1:c-myc* (NIT1ox) 69 homozygous lines were identified in subsequent generations. Of those 20 showed no significant transgene expression, 41 showed weak NIT1:c-myc expression and 8 showed substantial expression of the NIT1:c-myc construct. Western blots were utilized to compare the relative NIT1:c-myc content in the independent lines. From the latter group, three lines showing both stable and high level expression of recombinant NIT1:c-myc were selected for all further experiments. In case of the *35S::NIT2RNAi* (*NIT2*RNAi) lines, three independent lines with different knockdown efficiency were isolated.

### Metabolite analysis

The GC-MS/MS analysis of free IAA as well as conjugated IAA was carried out as previously described (Müller et al., 1998, 2002, 2006). In brief, the samples were pre-cleaned by micro-scale solid phase extraction on custom-made cartridges containing a silica-based aminopropyl matrix. Thereafter, the samples were taken to dryness, re-dissolved in 20 μL methanol, and treated with ethereal diazomethane. Subsequently samples were transferred to autosampler vials and excessive diazomethane and solvent was removed in a gentle stream of nitrogen. The methylated samples were then taken up in 15 μL of chloroform. In case of IAN, the mass spectrometric analysis was carried out following the description published by Müller et al. (1998). Here, indolic compounds are separated by HPLC prior to GC-MS analysis. Aliquots of 1 μL of each sample were injected into the GC-MS system for separation and mass fragment analysis using the autosampler and system quoted in the following. The IAA spectra were recorded on a Varian Saturn 2000 ion-trap mass spectrometer connected to a Varian CP-3800 gas chromatograph equipped with a CombiPal autoinjector (Varian, Walnut Creek, CA, USA). IAN spectra were recorded on a Finnigan MAT Magnum ion trap mass spectrometer connected to a Varian GC-3400 gas chromatograph equipped with an A200S autosampler (Finnigan MAT, Bremen, Germany).

### Root growth assays

For root growth assays, seeds were sterilized and plated onto Petri dishes containing ½-MS medium supplemented with 1% (w/v) sucrose. After 6 days, the seedlings were transferred to vertical plates containing ½-MS medium (control) and ½-MS medium supplemented with rising amounts of either IAA or IAN, respectively. About 20 seedlings were used per replicate and three replicates were made for each treatment. Primary root elongation was measured after 10 days using the ImageJ software (Abràmoff et al., 2004).

### Plant protein extraction, immunoblot analysis, and NIT activity assay

Protein extracts from Arabidopsis were used for two kinds of experiments. On the one hand, the proteins were utilized in immunoblot analysis of transgenic lines. On the other hand, the extracts were employed to estimate the nitrilase activity in NIT1ox lines compared to appropriate controls, i.e., wild type (ecotype Col-0) and *nit1-3*. For the extraction, 30–100 mg of plant tissue was frozen in micro-reaction tubes in liquid nitrogen before the tissue was disrupted using micro pestles (Sigma, Munich, Germany). As the mixture started to thaw, buffer (50 mM Tris/HCl, pH 7.4, 5 mM MgSO_4_, 1 mM EDTA, 1 mM EGTA, 1 mM PMSF, 5 mM DTT, 0.1% (v/v) Triton X-100, 10% (v/v) glycerol) was added in a ratio of 1:3 (w/v), and the suspension was further homogenized until no more tissue fragments were visible. Thereafter, cell debris and insoluble matter was collected by centrifugation (16,000 g, 15 min, 4°C), and the supernatant was transferred into fresh tubes. Protein concentrations were determined according to Bradford (1976) using serum albumin as a protein standard.

Both SDS-PAGE and immunoblot analyses of crude protein extracts followed standard procedures (Sambrook et al., 1998). Either a rabbit antiserum raised against native NIT1 (Schmidt et al., 1996) or a monoclonal α-c-myc IgG, clone 9.E10 (Evan et al., 1985), was used as first antibody in immunoblot analysis at a final dilution of 1:10,000 and 65 mg L^−1^, respectively. As second antibody goat-anti-rabbit-IgG (Promega, Madison, USA) or rabbit-anti-mouse-IgG (Promega Madison, USA), both coupled with an alkaline phosphatase, were used.

To quantify the nitrilase activity in crude protein extracts prepared from either bacteria or plants, reaction mixtures containing 200 μg protein and 5 mM IAN as substrate were analyzed in accordance to a previously described method (Pollmann et al., 2002). Although it has previously described that IAN is not the preferred substrate of nitrilases *in vitro* (Vorwerk et al., 2001), we selected IAN as substrate, because it is the only substance that serves as a potential precursor for a bioactive metabolite. The enzymatically produced IAA was detected photometrically at a wavelength of 280 nm after HPLC separation.

### Statistical analysis

The data were analyzed using either Student's *t*-test when two means were compared or one-way ANOVA followed by Tukey's B *post-hoc* test to allow for comparisons among all means. Statistical analyses were conducted using PRISM version 5.03 (GraphPad Software, http://www.graphpad.com/).

## Results

### Analysis of transcriptional perturbations of auxin biosynthesis-related genes through a blockade of the indole-3-acetaldoxime pathway

Previous work suggested the operation of nitrilases downstream of glucobrassicin (Barlier et al., 2000; Bak et al., 2001; Sugawara et al., 2009). This hypothesis was further confirmed by a whole-genome DNA microarray-based assessment that provided evidence for the existence of a transcriptional link between *SUR2* and *NIT1* and *NIT2*, respectively. The presented microarray data demonstrated that the expression of both *NIT1* and *NIT2*, but not *NIT3* is substantially down regulated in the *sur2* mutant (Morant et al., 2010). In order to gain a broader impression on the role of the IAOx pathway in Arabidopsis and to resolve how mutant plants cope with the loss of this pathway, a global analysis was performed to gain an ample overview of the biological processes perturbed in *cyp79b2*/*cyp79b3*. Herein, we were particularly interested in transcriptional changes of genes known to be involved in auxin biosynthesis, transport, and signaling. To disclose alterations in the global transcriptome, the gene expression pattern of the *cyp79b2*/*cyp79b3* mutant was compared to that of wild-type seedlings and monitored employing the Affimetrix ATH1 genome array containing more than 22,500 different 25-mer oligonucleotide probes representing approximately 24,000 gene sequences (Supplemental Data Sheet 1). Based on an arbitrary threshold of a *log*2 ratio ≥ 1.2 in combination with a *p*-value of 0.05, 620 genes were selected as differentially expressed in *cyp79b2*/*cyp79b3*. From these 620 genes 272 appeared to be up regulated, while 348 were significantly down regulated. Expectedly, both *CYP79B2* and *CYP79B3* were identified among the top-ranked down-regulated genes, due to the lack of these transcripts in the double mutant. To resolve which biological processes are mostly affected in the mutant, the two groups of up- and down-regulated genes were sorted with respect to their putative involvement in different biological processes using the gene ontology program BiNGO (Maere et al., 2005). BiNGO uses the available information in Gene Ontology Annotations (GO) and computes significant changes in the representation of those groups. Separately performed BiNGO analyses of the up- and down-regulated genes clearly revealed that genes in a small number of specific processes and cellular responses are significantly over-represented in *cyp79b2*/*cyp79b3*, whereas more processes appear to be significantly under-represented (Supplemental Data Sheet 2, Supplemental Presentation 1). The tested up-regulated genes were found to fall into three major groups, including stress responses, such as the defense responses to fungi and responses to abiotic stresses like light, salt and cold, responses to chemical stimuli, like, e.g., sucrose, fructose, glucose and chitin, and biological processes related with the catabolism of carboxylic acids, such as gibberellins. When examining the under-represented groups, expectedly, metabolic processes related with secondary metabolites, including the biosynthesis of glycosinolates, glucosinolates and phenylpropanoids became evident. Along with these processes, cell wall modification related processes could be detected. Most interesting, however, was the finding that many plant hormone-related processes, such as the responses to salicylic acid (SA), ethylene (ET), abscisic acid (ABA), jasmonic acid (JA), and GA seem to be altered in *cyp79b2*/*cyp79b3*, which suggests a central role in integrating and processing plant hormone crosstalk for the IAOx pathway. However, neither among the over- nor under-represented genes there was an enrichment of directly auxin-related gene groups detectable. Thus, we asked the question whether and to what extent the expression of known auxin-related genes is affected through the mutations in the *cyp79b2*/*cyp79b3* double mutant. Surprisingly, nearly no significant changes in the IPyA pathway were determined (Table 1). Out of the 14 putative genes involved in the IPyA pathway only *TAR2* and *YUC5* displayed a significant alteration in their levels of expression. The changes, however, were not consistent with an up-regulation of the IPyA pathway in response to the loss of the IAOx pathway, because *TAR2* expression appeared repressed, while *YUC5* transcript levels were elevated. In line with the results presented by Morant et al. (2010), expression of the nitrilases *NIT1* and *NIT2* was significantly suppressed in *cyp79b2*/*cyp79b3*. On the contrary, the expression of some other auxin biosynthesis-related genes, namely *SUR2, AtSP5a*, and *AMI1*, was found induced in the mutant. As pointed out before, the levels of IAA in the *cyp79b2*/*cyp79b3* double mutant are not affected (Zhao et al., 2002; Sugawara et al., 2009). Here we show that the reason for this is most likely not the compensation of the loss an auxin biosynthetic pathway by other metabolic routes that lead to IAA, but rather that the IAOx pathway does not contribute to general auxin biosynthesis under normal growth conditions. However, the experiments provided evidence for an involvement of the IAOx pathway in numerous stress responses, which gives reason to assume that this pathway is activated under particular circumstances, providing extra auxin if required for adequate responses to environmental stress factors. Intriguingly, we detected the induction of a small number of genes that contribute to Trp biosynthesis, indicating an enhanced metabolic activity of this route. In addition, we found the induction of two Gretchen Hagen 3 genes that are assumed to be involved in maintaining auxin homeostasis by conjugating excess auxin to amino acids. Together, these findings support the notion of enhanced auxin production. However, when checking auxin signaling-related gene, we found the prevailing induction of several IAA/Aux transcriptional repressors, i.e., *IAA2, IAA6, IAA19*, and *IAA29*, which was accompanied by the repression of some auxin response factors, i.e., *ARF3, ARF8, ARF11, ARF12*, and *ARF17*, suggesting reduced auxin signaling, which in most cases comes along with reduced free IAA contents (Table 1).

**Table 1:** **Differential expression of auxin biosynthesis-related genes in the ***cyp79b2***/***cyp79b3*** double mutant^a^**.

**Name**	**Gene ID**	***p*****-value**	***log*****2 ratio**
**TRYPTOPHAN BIOSYNTHESIS**			
ASA1^*^	At5g05730	4.51E-02	+1.06
TSA1^*^	At3g54640	5.76E-03	+0.88
TSB2^*^	At4g27070	1.67E-02	+0.62
**IPyA PATHWAY**			
*TAA1*	At1g70560	3.83E-01	−0.13
*TAR1*	At1g23320	6.37E-01	+0.05
TAR2^*^	At4g24670	2.17E-04	−0.69
*YUC1*	At4g32540	3.21E-01	−0.08
*YUC2*	At4g13260	2,74E-01	+0.22
*YUC3*	At1g04610	1.08E-01	−0.17
*YUC4*	At5g11320	5.48E-01	+0.06
YUC5^*^	At5g43890	3.61E-02	+0.93
*YUC6*	At5g25620	1.51E-01	−0.25
*YUC7*	At2g33230	5.86E-01	−0.08
*YUC8*	At4g28720	1.27E-01	+0.59
*YUC9*	At1g04180	2.62E-01	+0.47
*YUC10*	At1g48910	2.63E-01	−0.09
*YUC11*	At1g21430	1.91E-01	−0.12
**IAOx PATHWAY**			
CYP79B2^*^	At4g39950	2.96E-06	−4.86
CYP79B3^*^	At2g22330	3.52E-05	−2.98
**CAMALEXIN BIOSYNTHESIS**			
*CYP71A13*	At2g30770	1.23E-01	−0.45
NIT1/NIT2^*^	At3g44300	1.00E-03	−0.82
*NIT3*	At3g44320	8.59E-01	−0.03
*PAD3*	At3g26830	8.65E-01	−0.07
**INDOLE GLUCOSINOLATE BIOSYNTHESIS**			
*SUR1*	At2g20610	5.57E-02	−0.35
SUR2^*^	At4g31500	8.86E-03	+1.08
*UGT74B1*	At1g24100	9.58E-01	+0.02
AtSP5a^*^	At1g74100	3.77E-02	+0.78
**IAM PATHWAY**			
AMI1^*^	At1g08980	1.21E-02	+0.87
**GENES INVOLVED IN AUXIN CONJUGATION/DECONJUGATION**			
*IAR4*	At1g24180	7.99E-01	+0.02
*ILL1*	At5g56650	2.71E-01	−0.14
ILL3^*^	At5g54140	2.43E-02	+0.22
*ILL5*	At1g51780	1.43E-01	+0.78
*ILR1*	At3g02875	1.68E-01	+0.17
*UGT84B1*	At2g23260	6.67E-01	−0.03
IAGLU^*^	At4g15550	2.23E-03	−1.08
GH3.3^*^	At2g23170	1.86E-04	+2.07
GH3.4^*^	At1g59500	3.01E-03	+1.21
**AUXIN TRANSPORT**			
AUX1^*^	At2g38120	2.53E-02	+0.34
LAX1^*^	At5g01240	1.15E-02	+0.42
*LAX2*	At2g21040	7.62E-01	+0.04
LAX3^*^	At1g77690	1.67E-02	−0.35
*PGP1*	At1g36910	3.48E-01	+0.08
*PGP19*	At3g28860	6.38E-01	+0.06
*PIN1*	At1g73590	5.92E-01	−0.04
*PIN2*	At5g57090	1.69E-01	−0.20
*PIN3*	At1g70940	5.59E-01	+0.23
*PIN4*	At2g01420	7.97E-01	−0.05
PIN5^*^	At5g16530	1.05E-02	−0.45
*PIN6*	At1g77110	4.97E-01	−0.08
*PIN7*	At1g23080	5.06E-01	−0.15
*PIN8*	At5g15100	6.31E-01	−0.05
**AUXIN SIGNALING**			
*TIR1*	At3g62980	4.00E-01	−0.11
*AFB1*	At4g03190	3.45E-01	+0.26
*AFB2*	At3g26810	3.07E-01	−0.14
AFB3^*^	At1g12820	5.81E-03	−1.00
*AFB4*	At4g24390	6.60E-01	+0.04
*AFB5*	At5g49980	5.84E-01	−0.08
*ABP1*	At4g02980	5.91E-02	−0.25
*IAA10*	At1g04100	3.84E-01	+0.07
*SHY2*	At1g04240	1.41E-01	+0.45
AXR3^*^	At1g04250	6.97E-04	−0.75
IAA12^*^	At1g04550	1.58E-03	−0.63
*IAA34*	At1g15050	9.32E-01	−0.01
*IAA5*	At1g15580	1.36E-01	+0.97
*IAA18*	At1g51950	3.76E-01	+0.12
IAA6^*^	At1g52830	2.01E-04	+1.20
*IAA32*	At2g01200	4.44E-01	+0.05
*IAA8*	At2g22670	7.42E-02	−0.24
*IAA13*	At2g33310	4.40E-01	+0.11
*IAA20*	At2g46990	3.83E-01	+0.06
*IAA16*	At3g04730	7.94E-01	+0.04
IAA19^*^	At3g15540	8.58E-05	+1.77
*PAP1*	At3g16500	9.42E-01	−0.01
*IAA31*	At3g17600	3.72E-01	+0.07
IAA2^*^	At3g23030	7.79E-03	+0.85
IAA7^*^	At3g23050	9.47E-04	+0.52
*IAA30*	At3g62100	1.63E-01	+0.21
IAA14^*^	At4g14550	1.31E-02	−0.66
*IAA1*	At4g14560	8.95E-01	+0.04
*IAA11*	At4g28640	6.98E-01	+0.03
*PAP2*	At4g29080	6.49E-01	+0.03
IAA29^*^	At4g32280	3.01E-04	+2.72
*IAA28*	At5g25890	1.50E-01	−0.42
*AtAUX2-11*	At5g43700	7.63E-01	−0.10
*IAA9*	At5g65670	1.48E-01	+0.13
*ARF1*	At1g59750	3.36E-01	+0.09
*ARF2*	At5g62000	9.34E-01	−0.03
ARF3^*^	At2g33860	2.81E-03	−0.45
*ARF4*	At5g60450	1.86E-01	−0.23
*ARF5*	At1g19850	3.59E-01	−0.16
*ARF6*	At1g30330	9.47E-01	+0.01
*ARF7*	At5g20730	1.64E-01	+0.01
ARF8^*^	At5g37020	5.52E-03	−0.62
*ARF9*	At4g23980	1.96E-01	−0.12
*ARF10*	At2g28350	1.42E-01	−0.35
ARF11^*^	At2g46530	2.53E-03	−0.77
ARF12^*^	At1g34310	3.82E-02	−0.63
*ARF13*	At1g34170	1.06E-01	−0.15
*ARF16*	At4g30080	4.87E-01	−0.16
ARF17^*^	At1g77850	1.30E-02	−0.32
*ARF18*	At3g61830	2.14E-01	+0.38
*ARF19*	At1g19220	6.13E-02	+0.17
*ARF21*	At1g34410	4.93E-01	+0.04
*ARF23*	At1g43950	9.05E-01	+0.01

a Asterisks indicate significant differences between the corresponding gene expression in Col-0 and in cyp79b2/cyp79b3 (

* *p < 0.05; Student's t-test)*.

### Generation of gain- and loss-of-function lines

**Figure 2:**
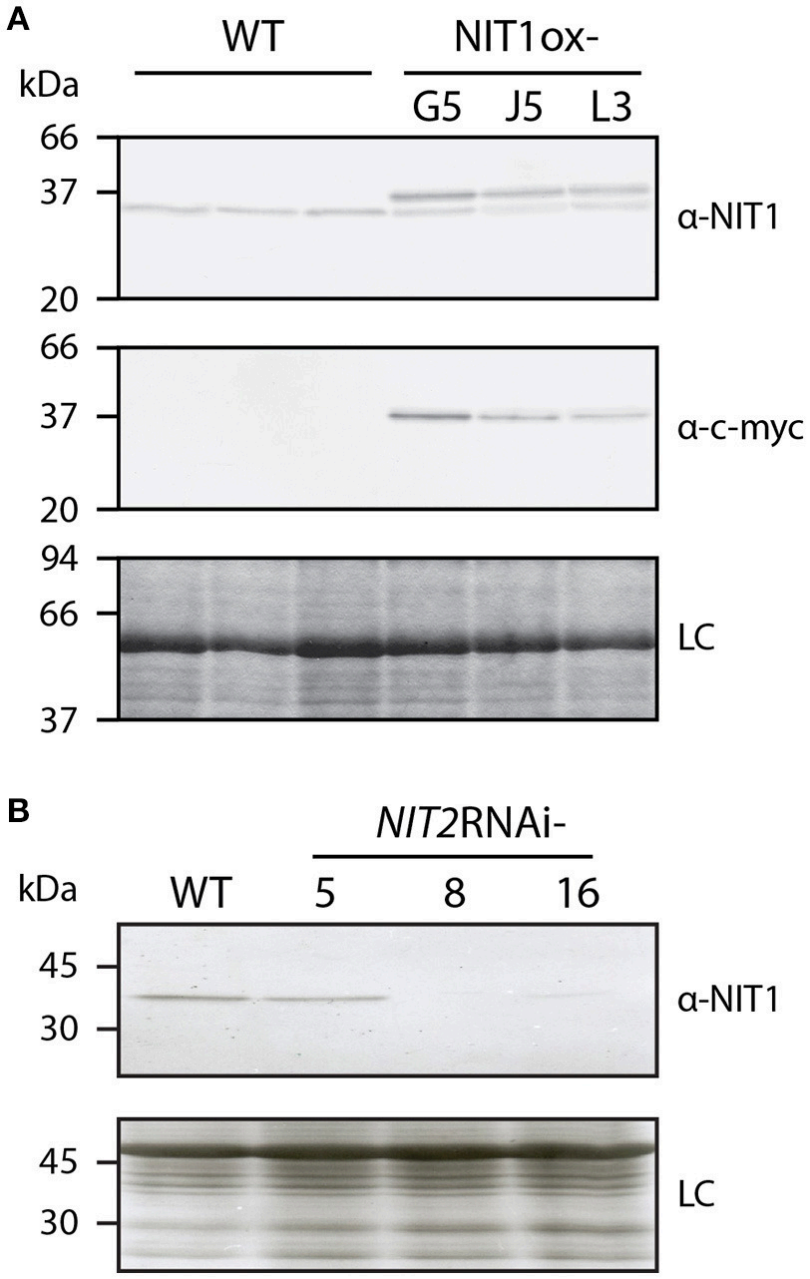
**Altered NITRILASE levels in homozygous NIT1ox and ***NIT2***RNAi lines**. The protein samples of leaf tissue (40 μg each) of 6–8 week-old plants were separated on 12.5% SDS polyacrylamide slap gels. **(A)** Three individual plants of *A. thaliana* (WT) and three NIT1ox lines were tested for their nitrilase expression pattern. The top panel shows the analysis with the α-NIT1 serum, while western blot analysis in the middle panel was carried out using a monoclonal α-c-myc antibody. The bottom panel shows a Coomassie blue-stained gel, indicating even loading (loading control, LC). **(B)** The nitrilase expression of three transgenic *NIT2*RNAi plants was compared to Arabidopsis wild type (WT). The upper panel shows a western analysis using a α-NIT1 antiserum, while the lower panel shows a Coomassie stained gel, indicating uniform loading (loading control, LC).

The conducted microarray analysis did not provide considerable evidence supporting a general role of the IAOx pathway in auxin formation. However, based on publicly available expression data, *NIT1* is not only expressed under stress conditions, but rather shows a broad expression pattern throughout plant development (Winter et al., 2007). Moreover, it has been demonstrated that *NIT1* is likely to contribute to lateral root formation in Arabidopsis (Müller et al., 1998). These phenomena made it tempting to speculate that NIT1 may possess a more general role in plant growth and development, rather than being restricted to stress responses. In order to investigate the role of NIT1 in more detail, we decided to take a genetic approach, testing the impact of both the constitutive over-expression of the gene and the RNA interference (RNAi)-mediated knockdown of the entire NIT1-subfamily. To this end, we engineered a construct to produce c-myc epitope-tagged NIT1 under the control of a 35S promoter that facilitates the specific immunological detection of the recombinant protein in corresponding mutant plants. Prior to the generation of stable transformants, the enzymatic properties of the chimeric NIT1:c-myc protein were analyzed. As preliminary experiments showed that N-terminally Strep-tagged NIT1 considerably lost activity compared to both wild type and C-terminally Strep-tagged variants of the protein (data not shown), we fused the c-myc epitope to the C-terminus of NIT1. Thereby, the additional amino acids AAAEQKLISEEDLNGAA (c-myc epitope is underlined) were added. To prove the functionality of the fusion protein, the NIT1:c-myc fragment was cloned into an appropriate bacterial over-expression system (Raymond et al., 1990), expressed from there, and the enzymatic activity of the bacterial extracts was analyzed. The experiments displayed a strong expression of recombinant NIT1:c-myc, which was monitored by western blot analysis using a α-c-myc antibody, as well as an expected increase of nitrilase activity in the 6000 g pellet fraction of the extract compared to either empty vector controls or the 6000 g supernatant fraction (Supplemental Presentation 2). After this initial proof of general applicability, the above mentioned NIT1:c-myc fragment was introduced into a binary vector downstream of a cauliflower mosaic virus 35S promoter that was subsequently used to transform Arabidopsis plants. BASTA selection was used to select for transformed individuals. It was possible to obtain 69 BASTA resistant lines of which 61 lines showed either no or only a moderate expression of the transgene. From the remaining 8 individual lines that showed a clear over-expression of the NIT1 construct three lines have been selected for further analyses, i.e., NIT1ox-G3, -J5, and -L3. By using either a α-NIT1 serum (Schmidt et al., 1996) and a monoclonal α-c-myc antibody (Evan et al., 1985), we were able to distinguish between the endogenous nitrilase signal and the recombinant protein, as the additional amino acids cause a shift of the molecular weight of approximately 2 kDa (Figure 2).

Apart of the analysis of the consequences of the over-expression of NIT1, we aimed at the examination of plants that lack the activity of nitrilases of the NIT1-subgroup. The available *nit1-3* mutant shows, except of the increased tolerance against exogenously applied IAN, no significant changes compared to wild-type plants (Normanly et al., 1997), which is likely due to the redundant function and compensatory effect of the other nitrilase isoenzymes. The members of the NIT1-clade are located on chromosome 3 and organized in a *NIT2/NIT1/NIT3* gene cluster (Hillebrand et al., 1998), which renders the generation of higher order knockout mutants by conventional crossing basically impossible. However, the NIT1-clade isogenes share a very high sequence similarity. Hence, a transitive RNAi approach was applied to suppress the expression of the complete isogene family (Sidahmed and Wilkie, 2010). For this, an appropriate construct, comprising a highly conserved cDNA fragment, was cloned and introduced into *A. thaliana*. We were able to identify three individual BASTA resistant lines, which showed differential expression of nitrilases, as assayed by western blot analysis using a α-NIT1 antiserum that has been shown to cross-react with all members of the NIT1-clade (Vorwerk et al., 2001). Out of the identified lines *NIT2*RNAi-8 showed the strongest RNAi effect, being practically free of any immunological signal. In contrast, line *NIT2*RNAi-5 displayed only a very weak if any repression of the nitrilases, while *NIT2*RNAi-16 showed a very fade remaining nitrilase signal (Figure 2). Thus, lines 8 and 16 were used in all following experiments.

### Phenotypic analysis of *NIT1* mutant lines

**Figure 3:**
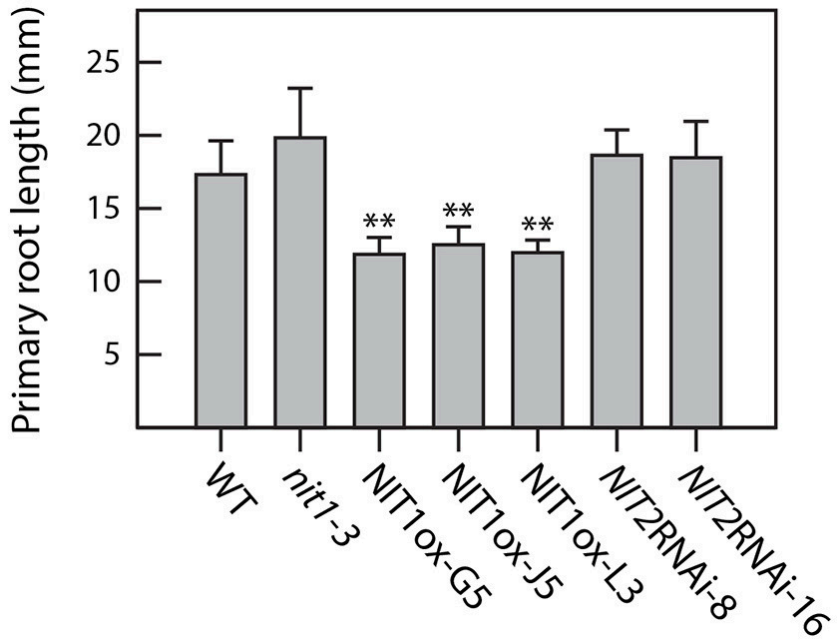
**Primary root elongation of NIT1ox compared to ***nit1-3***, NIT2RNAi, and wild-type seedlings**. Root elongation was quantified in 2 week-old seedlings grown on vertical plates. Seedlings were removed from the Petri dishes, and root length was recorded. At least 15 seedlings of each genotype were measured for each condition. Error bars indicate the standard error of the mean. Asterisks indicate significant differences between Col-0 and the different tested genotypes (^**^p < 0.001; Student's *t*-test).

**Figure 4:**
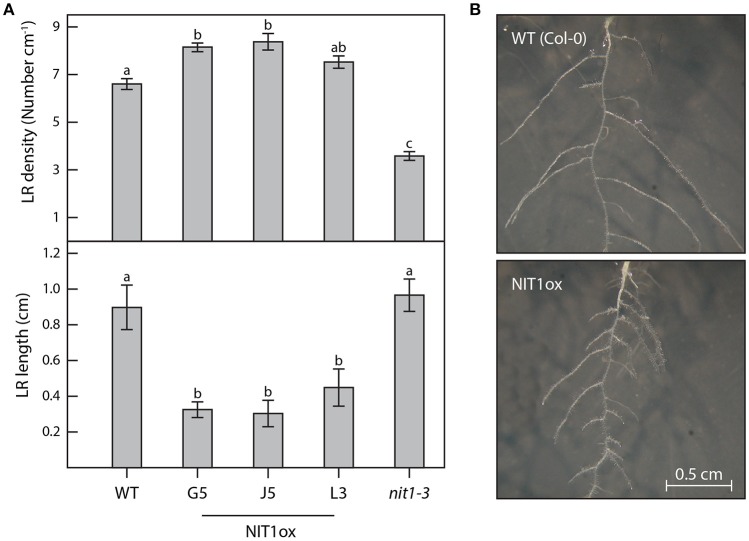
**Differences in root architecture between wild type, NIT1ox, and ***nit1-3*** mutants**. Two week-old plants grown in vertical surface culture on solidified ½ MS medium were analyzed for alterations of their root morphology. **(A)** For the analysis of lateral root (LR) density and length a minimum of 12 individual plants per genotype were studied. Error bars indicate the standard error of the mean. Different letters indicate significant differences between means (*p* < 0.05). **(B)** The images show magnifications of the root systems of wild-type plants (WT) and NIT1ox for better perceptibility. The images are scaled to the same size.

Compared to wild-type plants, neither NIT1ox lines nor the *NIT2*RNAi lines showed significant differences in growth habit when examining overground organs of 6–9-week old plants. Taking into account that NIT1 has previously been described to be capable of converting IAN to IAA, this finding was unexpected as the accumulation of NIT1:c-myc has been shown in protein extracts of the examined NIT1ox plants (Figure 2). In contrast, the root phenotype of young NIT1ox seedlings differed remarkably from that of wild-type plants, *nit1-3* knockout mutants, and the *NIT2*RNAi lines, respectively. The NIT1ox plants exhibited a strong reduction in primary root length (Figure 3), whereas the primary roots of the *nit1-3* mutant and of the two *NIT2*RNAi lines resembled the wild type. A closer inspection of the root morphology revealed that in NIT1ox the number of lateral roots slightly increased, while the lateral roots are apparently shorter (Figure 4A). In addition, the amount of root hairs in the NIT1ox line is substantially higher relative to the wild type (Figure 4B).

**Figure 5:**
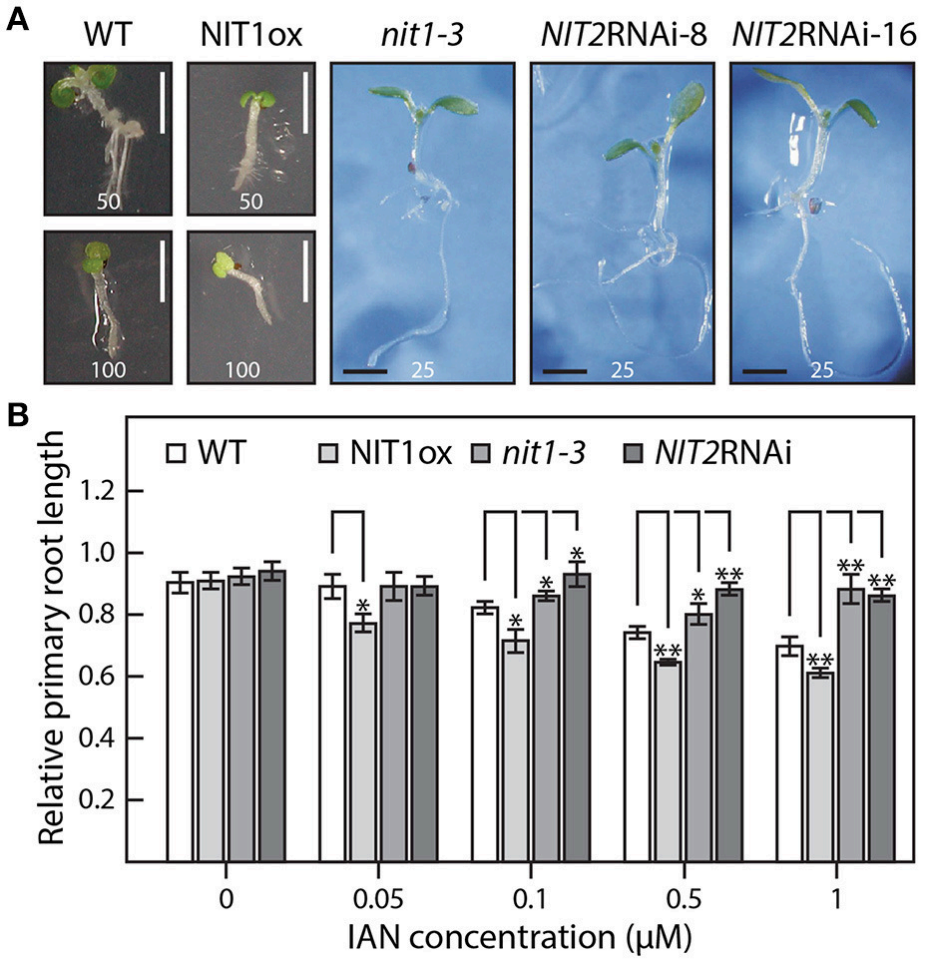
**IAN effects on the wild type as well as on NIT1ox, ***nit1-3***, and ***NIT2***RNAi plants. (A)** Seven day-old seedlings grown under sterile conditions with the indicated amounts of IAN in the media given in μM. Scale bar = 2 mm. **(B)** Assessment of the differential effect of IAN on root elongation in the studied genotypes. In order to examine the sensitivity toward IAN in the media without the bias of initially shorter primary roots, the influence of IAN is expressed in relative terms. To do so, the length of the longest primary root of each genotype grown under control conditions was set to a value of 1 and all other roots of the corresponding genotype were expressed relative to this value. The concentration of the IAN content in the plates is indicated. For each condition and genotype at least 15 seedlings were measured. The data represent means ± SE. Asterisks indicate significant differences between the corresponding WT control and the different tested genotypes under the given condition (^*^p < 0.05, ^**^p < 0.01; Student's *t*-test).

At first sight, the phenotype of the knockdown mutants was virtually the same as described for the functional knockout line, *nit1-3*. Despite looking quite like wild type under normal growth conditions, the knockdown line showed the expected increased tolerance toward IAN in the solidified growth medium, while showing wild type reactions when grown on medium containing IAA. A more detailed analysis, however, revealed some slight alterations compared to *nit1-3*. Line *NIT2*RNAi-8 appeared to tolerate even higher IAN concentrations and showed a reduced responsiveness toward IAA in the media (Figure 5B, Supplemental Image 1). At the same time, the NIT1ox plants behaved hypersensitive to both exogenous IAA and IAN. Especially at high IAN concentrations, the NIT1 over-expressing plants were significantly impaired in growth, whereas *nit1-3* as well as the two *NIT2*RNAi lines appeared fairly insensitive to the IAN in the media (Figure 5).

Taking together, this observations led us to conclude that IAA levels in NIT1ox lines are likely to be higher than in wild type, resulting in the observed additive auxin effect.

### Analysis of nitrilase activity in NIT1ox plants

**Figure 6:**
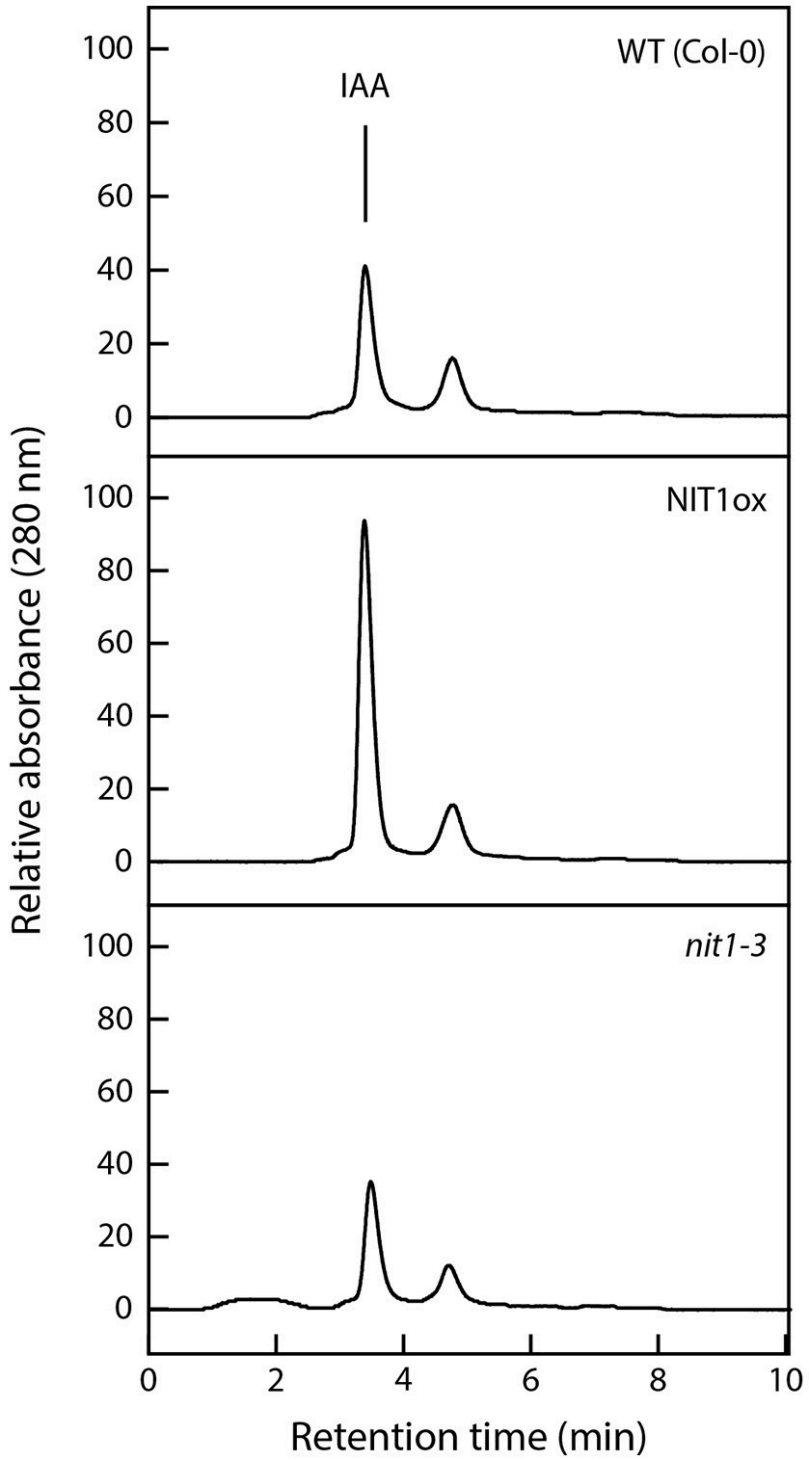
**Analysis of nitrilase activities in protein extracts of wild type, NIT1ox, and ***nit1-3*** mutants**. Crude extracts (200 μg) were prepared from 2 week-old seedlings grown on ½ MS media. Samples were incubated overnight at 30°C with 5 mM IAN as substrate. The enzymatically produced IAA was analyzed photometrically at a wavelength of 280 nm after separation by HPLC.

To corroborate that the severe phenotypic alterations in NIT1ox are indeed caused by an increase of nitrilase activity in the seedlings, enzyme activity was determined in the extracts from the same plantlets that have been used for the root elongation assay. The data shown in Figure 6 demonstrate that NIT1ox plants, which have a higher steady-state level of NIT1, also displayed an approximately 2.3-fold higher enzyme activity. This is reflected by higher levels of the reaction product, IAA, eluting at 3.4 min. Intriguingly, the extracts from *nit1-3* showed only a reduction of nitrilase activity of approximately 15% relative to the wild type. This is on the one hand astonishing as NIT1 is the isoenzyme with the highest expression level *in planta*, on the other hand this might implicate that a loss of NIT1 can partially be compensated by the remaining nitrilase isoenzymes.

### Auxin analysis

After the phenotypic analysis, we decided to analyze the chemotype of the investigated genotypes in closer detail. In a first approach, we compared IAA and IAN levels in 6–8 week-old plants by mass spectrometry. The obtained data confirmed the absence of phenotypic differences of overground organs of plants of this developmental stage, because they neither showed significant changes of free and total IAA levels nor of IAN in the NIT1ox mutant compared to wild type (Table 2). Although the plants were proven to accumulate considerable amounts of enzymatically active, recombinant NIT1, the IAN contents in the leaf material appeared to be reduced by only around 700 pmol (g FW)^−1^, thus, lying within the range of the error of the detection method. It can be concluded that under these conditions the levels of IAN are likely too low for an efficient conversion to IAA by the recombinant nitrilase.

**Table 2:** **IAA and IAN contents in 6–8 week old wild type and transgenic NIT1ox plants^a^**.

**Plants**	**Free IAA**	**Total IAA**	**IAN**
Wild type	0.02 ± 0.002	9.2 ± 1.3	1.7 ± 0.3
NIT1ox	0.02 ± 0.003	6.8 ± 3.2	1.0 ± 0.5

a *The values are given in nmol (g FW)^−1^. Shown are means ± SD from three independent experiments*.

However, the analysis of auxin contents in sterile grown seedlings, which show phenotypic differences, revealed very intriguing aspects. Most likely being the reason for the aberrant root phenotype, the NIT1ox line displayed clearly elevated levels of both free IAA and IAN (Table 3), while the total IAA pool remained unaffected. Free IAA was increased 2.3-fold (0.23 ± 0.03 nmol (g FW)^−1^ compared with 0.10 ± 0.01 nmol (g FW)^−1^; *n* = 3), and IAN was increased 3.5-fold (172 ± 18 nmol (g FW)^−1^ compared with 48 ± 13 nmol (g FW)^−1^; *n* = 3). Whereas the increase of IAA was expected, due to the over-expression of a nitrilase, which was already suspected to be able to convert IAN to IAA, the considerable increase of the IAN pools was unexpected. In conclusion, these results point toward an elevated release of IAN from secondary metabolites like, i.e., glucosinolates (for review see Grubb and Abel, 2006; Halkier and Gershenzon, 2006), in particular the putative IAN precursor glucobrassicin, or an activation of *de novo*-synthesis of IAN. This seems to be affected by the alteration of the NIT1 content *in planta* in an either direct or indirect fashion. Interestingly, neither *nit1-3* nor *NIT2*RNAi plants displayed significantly altered free IAA and IAN contents. However, the levels of total IAA are significantly decreased in these lines. This could be taken as a hint toward a nitrilase-dependent regulation of the total IAA pool in Arabidopsis.

**Table 3:** **Comparison of IAN and IAA contents in 2 week old seedlings of wild type, NIT1ox, ***nit1-3***, and ***NIT2***RNAi plants^a^**.

**Plants**	**Free IAA**	**Total IAA**	**IAN**
Wild type	0.10 ± 0.01	279 ± 8	48 ± 13
NIT1ox	0.23 ± 0.03^*^	319 ± 8	172 ± 18^**^
*nit1-3*	0.12 ± 0.02	154 ± 28^*^	88 ± 8
*NIT2*RNAi	0.11 ± 0.02	69 ± 19^**^	30 ± 4

a The values are given in nmol (g FW)^−1^. Shown are means ± SD from three independent experiments. Asterisks indicate significant differences in metabolite contents referred to the corresponding metabolite content in wild-type Arabidopsis (

* *p < 0.05*,

** *p < 0.01; Student's t-test)*.

## Discussion

Auxins are under investigation for more than 100 years and it has become common knowledge that indole-3-acetic acid (IAA), the major plant growth hormone, is involved in virtually all aspects of plant growth and development (Woodward and Bartel, 2005; Zhao, 2010). Although we already obtained a very good picture of the physiological functions and even the molecular mechanisms of auxin perception (Dharmasiri et al., 2005; Tan et al., 2007), our recent understanding of IAA biosynthesis and the control of cellular auxin homeostasis are still fragmentary. While it is generally accepted that the major portion of IAA is produced via the IPyA pathway (Stepanova et al., 2011; Won et al., 2011), several other routes are supposed to operate in plants either in a redundant manner or specifically induced by particular circumstances like biotic/abiotic stimuli and developmental processes, respectively (Pollmann et al., 2006; Zhao, 2014; Kasahara, 2015; Figure 1).

Nitrilases, which have been identified in Arabidopsis in the early nineties of the last century (Bartling et al., 1992, 1994; Bartel and Fink, 1994), were believed to play a key role in auxin biosynthesis for quite a long while. To date, several lines of evidence have casted reasonable doubts on this assumption. In this particular regard, the lack of a clear phenotype of the *nit1-3* mutant, except the reduced sensitivity to exogenous IAN (Normanly et al., 1997), and the *in vitro*-analysis of NIT1—NIT3 (Vorwerk et al., 2001; Jenrich et al., 2007), showing only a slow conversion of IAN into IAA, have to be mentioned. Nevertheless, this slow hydrolysis of IAN showed to be sufficient to produce a clear auxin over-expression phenotype in Arabidopsis if IAN was applied exogenously (Schmidt et al., 1996; Normanly et al., 1997). There are a couple of publications emphasizing a rather more specific function for nitrilases of the NIT1-subfamily, helping to activate auxiliary pathways of IAA synthesis under particular circumstances. It has been shown that *NIT1* as well as *NIT2* expression is induced in the course of bacterial infection with *Plasmodiophora brassicae* (Grsic-Rausch et al., 2000). Furthermore, induction of *NIT3* gene expression has been described to occur under sulfate-starvation, which in turn was accompanied by an increase of nitrilase activity in cell-free extracts, and obvious alterations of the root morphology. At the same time the glucosinolate contents were demonstrated to drop (Kutz et al., 2002; Nikiforova et al., 2003; Hirai et al., 2005). Taking into account that the degradation of glucobrassicin in Arabidopsis roots was detected to be faster than the conversion of other glucosinolates, a model was proposed in which the release of IAN from glucobrassicin and the subsequent conversion into IAA by the induced NIT3 isoenzyme stimulated root growth and lateral root formation, resulting in the penetration of new soil areas (Kutz et al., 2002). However, the physiological function of Arabidopsis NIT1 has not been elucidated beyond doubts so far.

In order to gain conclusive evidence on a putative transcriptional link between the major auxin biosynthetic pathway and the most likely crucifer-specific IAOx pathway, microarray analyses were conducted comparing transcriptional profiles of 14 day-old *cyp79b2*/*cyp79b3* and wild-type seedlings. Against our expectations, the experiments revealed no significant perturbation of genes involved in the IPyA pathway. If at all, an indole-3-acetamide (IAM)-dependent pathway, including AMI1, might contribute to the compensation of the loss of the IAOx pathway in in the *cyp79b2*/*cyp79b3* double mutant, because not all of the cellular IAM originates from IAOx and *AMI1* shows a significant induction. However, at present it is still not clear how IAM is synthesized, therefore a final conclusion on the influence of this pathway cannot be drawn beyond doubt. Hence, with respect to our current knowledge, it appears seemingly likely that IAOx-dependent processes do not play important roles in the basic formation of auxin. Much more, our results confirm the observation of several other groups, claiming a contribution of the IAOx-dependent processes in stress responses in Arabidopsis (Kutz et al., 2002; Nikiforova et al., 2003; Hirai et al., 2005; Morant et al., 2010). After not being able to provide unequivocal evidence for a contribution of the IAOx pathway to general IAA production under normal conditions, there was the remaining question why *NIT1* shows such a broad expression pattern. Hence, we decided to address the open question whether NIT1 may nonetheless contribute to developmental processes and IAA homeostasis under normal growth conditions. To this end, a genetic approach has been taken. Firstly, we constructed and analyzed NIT1 over-expressing *A. thaliana* mutants (NIT1ox). In this respect, we were able to determine a noteworthy alteration of root morphology of the mutant plants. Solely by increasing the total level of NIT1 in the plant, the primary root length was reduced by approximately 40% compared to wild-type Arabidopsis and nitrilase impaired mutant plants (*nit1-3, NIT2*RNAi) of the same age (Figure 3). The reduction of primary root length was accompanied by an increased number of shorter lateral roots and root hairs. Subsequent mass spectrometric analysis of metabolite contents revealed a significant change of auxin levels in the NIT1ox seedlings. The level of free IAA was increased by approximately 2.3-fold, while the IAN pool was shown to be around 3.5-fold higher. In summary, the metabolic changes evoked by NIT1 over-expression are clearly more pronounced than the results reported by Normanly et al. (1997) over-expressing NIT2. In agreement with these findings, crude extracts of NIT1ox seedlings showed an expected increase in nitrilase activity.

Based upon the data outlined above, the increased NIT1 content seems to be responsible for the aberrant cellular concentration of indolic constituents in the mutant, at least, in young seedlings, as this effect could not be detected at later developmental stages. Especially the increase of the IAN level is of particular interest. It has earlier been described that increased IAN levels are able to induce nitrilase transcription (Müller and Weiler, 2000), but that it works *vice versa* has not been described until now. In any case, the increased NIT1 expression in the NIT1ox line seems to be the cause for the activation of either IAN *de novo*-biosynthesis via indole-3-acetaldoxime (Slovin et al., 1999; Nafisi et al., 2007) or the release of IAN from plant secondary compounds, i.e., glucobrassicin.

Interestingly, *nit1-3* and NIT2RNAi plants show a severe reduction of the total IAA pool, while not exhibiting significant changes in levels of free IAA and IAN (Table 3). The content of total IAA was determined to be only around 25% of the contents measured for wild-type plants. Together with the considerably elevated levels of total IAA in NIT1ox, this may suggest that nitrilase-mediated IAA production is closely linked to auxin conjugation and deconjugation, respectively. Obviously, IAA production in *NIT2*RNAi seedlings is not significantly affected, which underlines the assumptions that (a) nitrilases are not vital for general IAA biosynthesis and (b) that redundant IAA pathways are likely to be operative *in planta*.

In fact, the point that the aberrant phenotype of NIT1ox is limited to roots of young seedlings is a very compelling aspect, distinguishing NIT1ox from other well documented Arabidopsis auxin over-producing mutants, like, e.g., *bus1-1* (Reintanz et al., 2001), *35S::CYP79B2* (Hull et al., 2000), and *yucca* (Zhao et al., 2001; Cheng et al., 2006). Here, the phenotypic changes are visible in roots as well as shoots, and leaves. This can most likely be due to three different reasons: (a) IAN, the proposed substrate of NIT1, could preferentially accumulate in root tissue reaching local concentrations that promote the hydrolysis by the recombinant nitrilase; (b) an increase of the physiologically active free IAA content is quickly compensated by conjugation to either sugars, proteins, or amino acids. Hence, aberrations are restricted to roots because they respond much more sensitive to alterations of the IAA pool (Thimann, 1938); and (c) NIT1 could undergo post-translational modification in a tissue specific fashion, which could lead to an altered affinity to its substrate. The latter aspect is emphasized by a couple of publications, demonstrating differential behavior of NIT1 in response to stress, i.e., the aggregation of NIT1 as an early event in wound and pathogen induced cell-death (Cutler and Somerville, 2005), and the stress-induced S-glutathionylation (Dixon et al., 2005). However, a variety of other post-translational modifications, like for instance phosphorylation, are also conceivable.

In summary, based on our results we can likely rule the functionality of NIT1 in general auxin biosynthesis in older plants out. However, at least our observations provide clear evidence for an operability of the enzyme in early stages of seedling development and here in root development in particular, converting IAN to IAA. Nevertheless, the increased IAN contents in NIT1ox plants and the significantly decreased total IAA levels in *nit1-3* and *NIT2*RNAi remain enigmatic. Considering that nitrilase activity not only results in the conversion of nitriles to their corresponding acids, but give also rise to amide by-products at a fairly constant ratio (Pollmann et al., 2002), IAM might represent the missing link to explain the observed phenomenon. In a preliminary series of experiments we tested this hypothesis and were able to detect a down-regulation of *NIT1* and *NIT2* expression by exogenously applied IAM. On the contrary, IAN was not found to induce the expression of *AMI1*. However, much more experiments are needed to unravel this seemingly more complex regulatory network. Further analysis of the contribution of nitrilases in the complex framework of auxin biosynthesis in Arabidopsis and their connection to other enzymes known to be involved in auxin production need to be investigated prior to draw a final conclusion. In this context, the generated RNAi lines can be of tremendous benefit, as they feature plant lines in which a pathway branch is significantly repressed. Crossings with other well-documented knockout lines, like for instance the *cyp79b2/cyp79b3* double mutant (Zhao et al., 2002) that supposedly act upstream of the nitrilases or an *ami1* knockout mutant (Aronsson et al., 2007) that is likely to compete with the nitrilases on the direction of the indole-3-acetaldoxime flux, could be of great scientific interest. Further investigation of the relationship between nitrilases and the major auxin biosynthesis pathway via TAA1 and the YUCCA proteins, however, does not seem to be exceptionally promising. Since the microarray data presented here demonstrate that a knockout of the entire IAOx pathway does not perturb the expression of genes involved in the major pathway for auxin biosynthesis, and because microarray analyses using *sav3* mutants (GSE9816) did not reveal any substantial impact on the expression of *NIT1*–*NIT3* (Tao et al., 2008), it has to be concluded that there is no coupling of the pathways on the transcriptionally level.

## Author contributions

SP and MP designed the research; TL, TJ, IT, BS, MMPA, and SP performed the research; TL, TJ, MP, IT, BS, MMPA, and SP analyzed the data; SP wrote and edited the manuscript.

## Funding

The presented work was funded by grants (SP: SFB480/A10, BFU-2014-55575-R; MP: SPP1152, PI424/2-1) from the Deutsche Forschungsgemeinschaft (DFG), Bonn, Germany, and the Spanish Ministry of Economy, Industry and Competitiveness (MINECO).

### Conflict of interest statement

The authors declare that the research was conducted in the absence of any commercial or financial relationships that could be construed as a potential conflict of interest.

## Supplementary material

Supplemental Data Sheet 1 **Microarray analysis of the ***cyp79b2***/***cyp79b3*** double mutant in comparison to wild-type Arabidopsis**. RNA from 2 week-old *cyp79b2*/*cyp79b3* and wild-type Arabidopsis seedlings was prepared and subjected to microarray analysis using affimetrix Ath1 genome chips. For each genotype three chips were hybridized and the data statistically analyzed employing the Limma package in R.

Supplemental Data Sheet 2 **BiNGO analysis of selected up- and down-regulated genes in ***cyp79b2***/***cyp79b3*****. After filtering the microarray data according to the arbitrary set values of a *p* ≤ 0.05 and a *log2* ratio > 1.2, gene enrichment and clustering in functional groups was analyzed using the BiNGO software package in Cytoscape 3.4.0. Over- and under-represented GO groups are given in the file.

Supplemental Presentation 1 **BiNGO analysis of selected up- and down-regulated genes in ***cyp79b2***/***cyp79b3*****. Graphical representations of over- and under-represented GO groups are provided in two separate figures.

Supplemental Presentation 2 **Functional expression of C-terminally c-myc-tagged NITRILASE 1 from ***Arabidopsis thaliana*****. The NIT1:c-myc fusion construct as well as an empty vector control (pEXP1) were expressed in *Escherichia coli* as outlined in *Material and methods*. Aliquots of 12.5 μg of protein were subjected to SDS-gel electrophoresis (12.5%) and immunoblotting. The top panel shows the Coomassie blue-stained gel, for the immunoblot (bottom) only the region around 38 kDa is shown. Lanes 1, 4: crude extracts; lanes 2, 5: 6000 g supernatants; lanes 3, 6: 6000 g pellets. Samples of each fraction (200 μg) were incubated overnight at 30 °C with 5 mM IAN as the substrate. The produced IAA was analyzed photometrically at a wavelength of 280 nm after separation by HPLC. The solvent used for HPLC separation consisted of aqueous formic acid (0.5 M)-saturated n-hexan: ethyl acetate (60:40, v/v).

Supplemental Image 1 **Primary root elongation of wild type, NIT1ox, ***nit1-3***, and ***NIT2***RNAi mutants grown on media containing either IAN or IAA**. Seedlings were grown on media containing risings amounts of IAN and IAA, respectively. Since the obtained transgenic lines for NIT1ox and *NIT2*RNAi showed no phenotypic differences, only data obtained for NIT1ox-J5 and *NIT2*RNAi-8 are shown for more clarity. The numbers in the pictures indicate the concentration of IAN and IAA in the used media given in μM. For each condition and genotype at least 15 seedlings were measured. Error bars indicate the standard error of the mean.

## Abbreviations

EMS: ethyl methansulfonate
HPLC: high performance liquid chromatography
IAA: indole-3-acetic acid
IAN: indole-3-acetonitrile
NIT: nitrilase
RNAi: RNA interference.
