# Supplementary material for: Arabidopsis NITRILASE 1 Contributes to the Regulation of Root Growth and Development through Modulation of Auxin Biosynthesis in Seedlings
Source: Front Plant Sci. 2017 Jan 24;8:36. doi: 10.3389/fpls.2017.00036 (PMC5258727; doi:10.3389/fpls.2017.00036)
Supplement: Supplemental Presentation 1 — BiNGO analysis of selected up- and down-regulated genes in cyp79b2/cyp79b3. Graphical representations of over- and under-represented GO groups are provided in two separate figures. [file Presentation1.PPTX]

## Slide 1
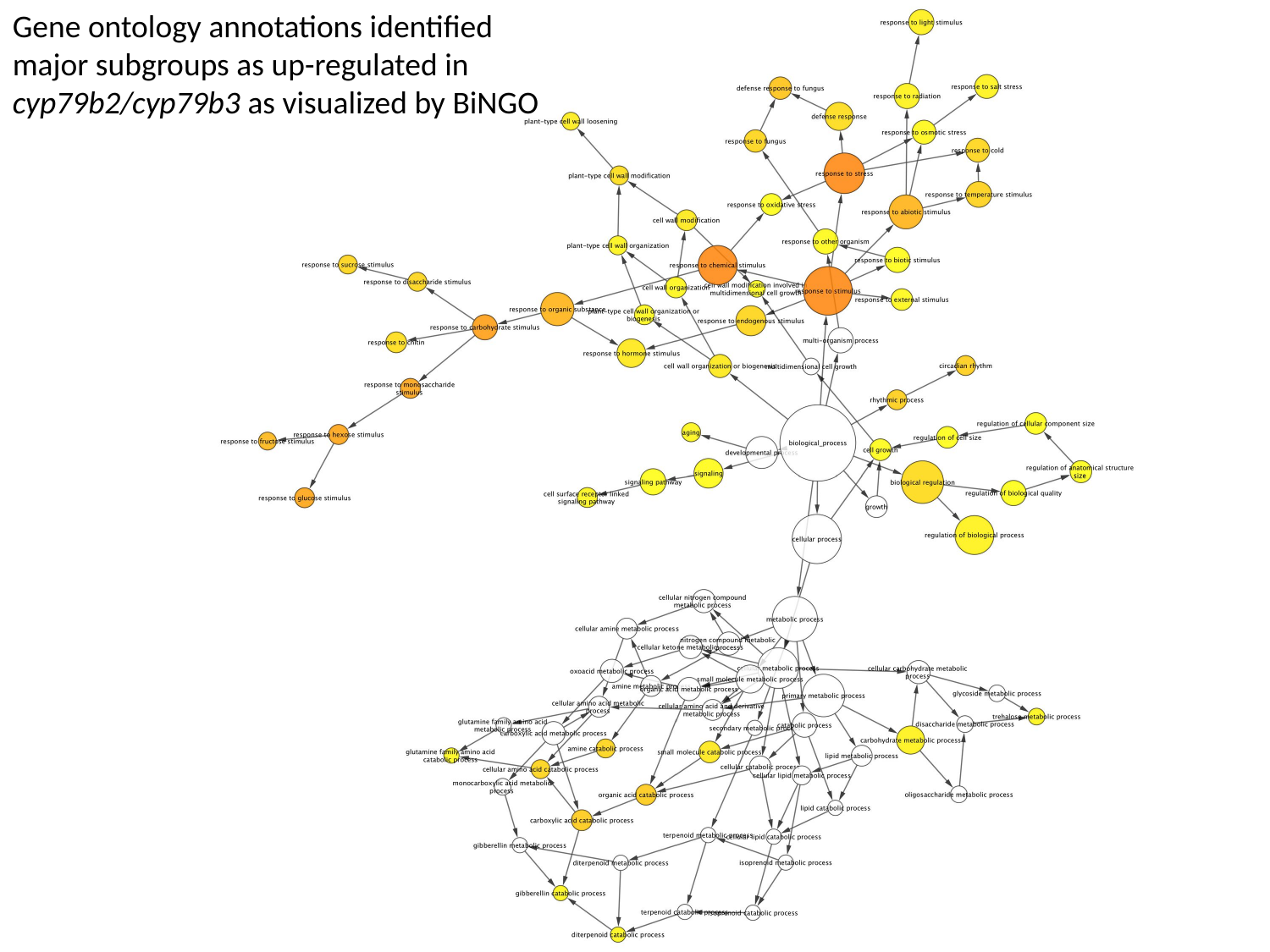

Gene ontology annotations identified major subgroups as up-regulated in cyp79b2/cyp79b3 as visualized by BiNGO

## Slide 2
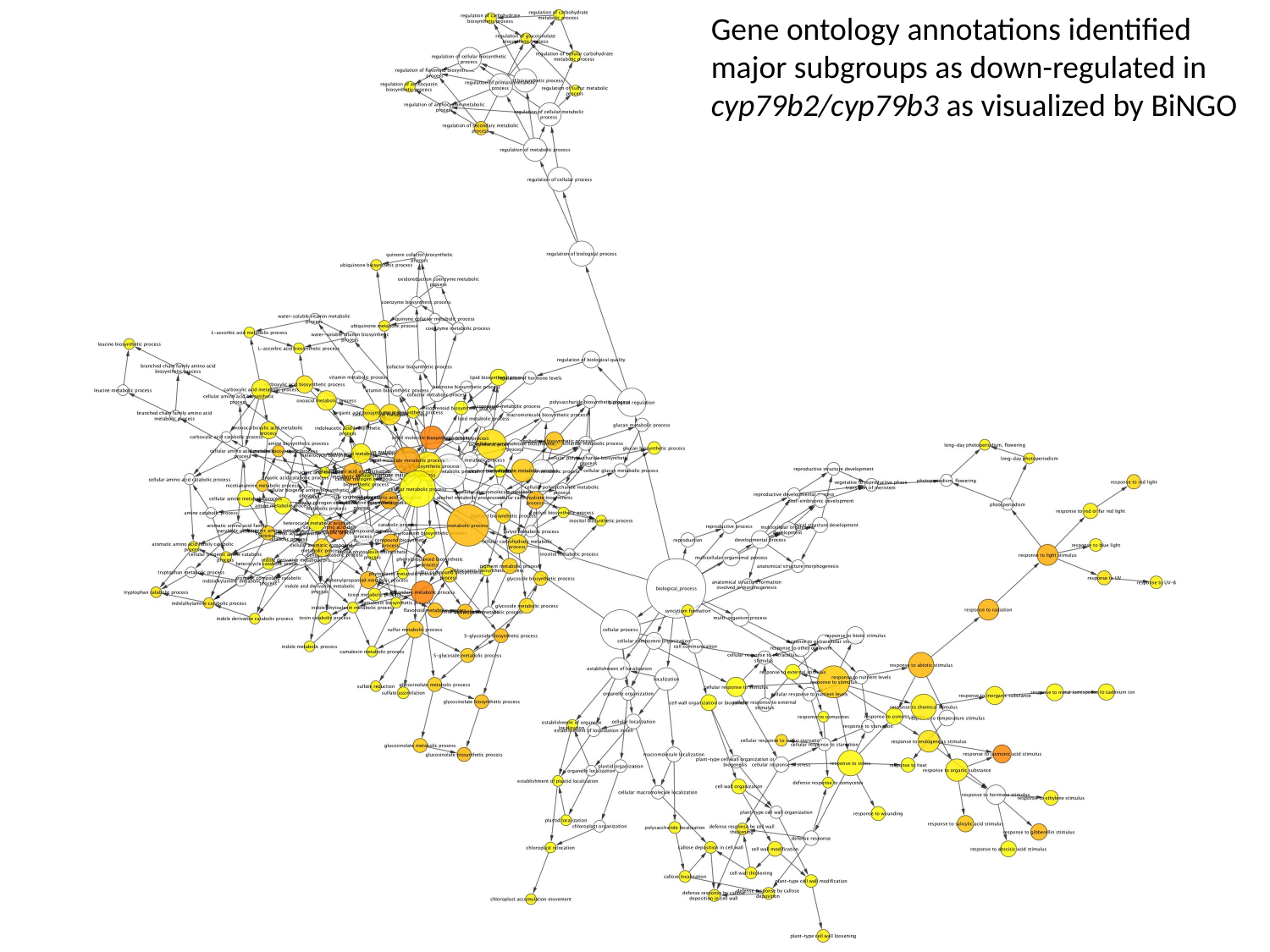

Gene ontology annotations identified major subgroups as down-regulated in cyp79b2/cyp79b3 as visualized by BiNGO
