# Supplementary material for: Arabidopsis NITRILASE 1 Contributes to the Regulation of Root Growth and Development through Modulation of Auxin Biosynthesis in Seedlings
Source: Front Plant Sci. 2017 Jan 24;8:36. doi: 10.3389/fpls.2017.00036 (PMC5258727; doi:10.3389/fpls.2017.00036)
Supplement: Supplemental Presentation 2 — Functional expression of C-terminally c-myc-tagged NITRILASE 1 from Arabidopsis thaliana. The NIT1:c-myc fusion construct as well as an empty vector control (pEXP1) were expressed in Escherichia coli as outlined in Material and methods. Aliquots of 12.5 μg of protein were subjected to SDS-gel electrophoresis (12.5%) and immunoblotting. The top panel shows the Coomassie blue-stained gel, for the immunoblot (bottom) only the region around 38 kDa is shown. Lanes 1, 4: crude extracts; lanes 2, 5: 6000 g supernatants; lanes 3, 6: 6000 g pellets. Samples of each fraction (200 μg) were incubated overnight at 30 °C with 5 mM IAN as the substrate. The produced IAA was analyzed photometrically at a wavelength of 280 nm after separation by HPLC. The solvent used for HPLC separation consisted of aqueous formic acid (0.5 M)-saturated n-hexan: ethyl acetate (60:40, v/v). [file Presentation2.PPTX]

## Slide 1
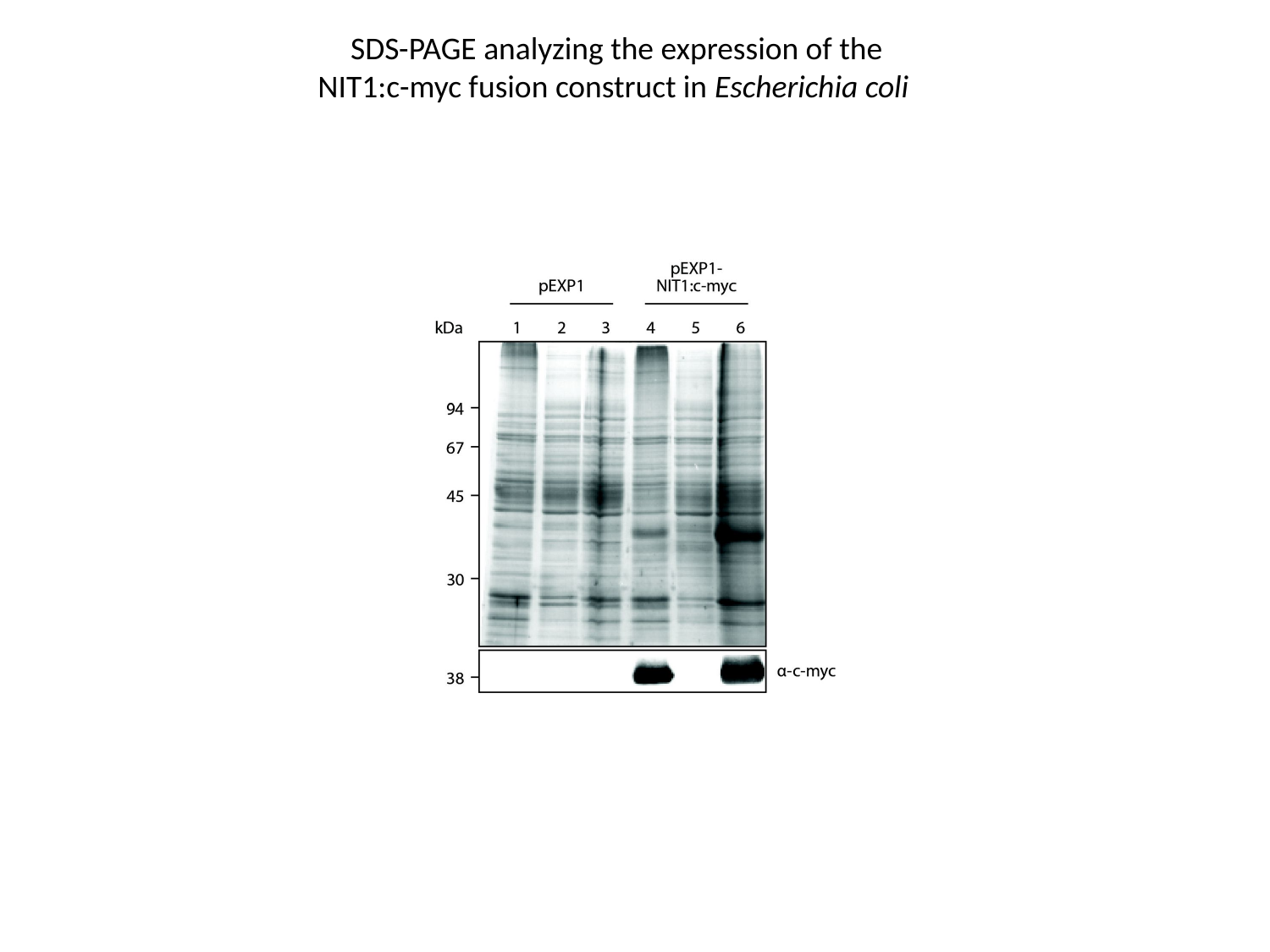

SDS-PAGE analyzing the expression of the NIT1:c-myc fusion construct in Escherichia coli

## Slide 2
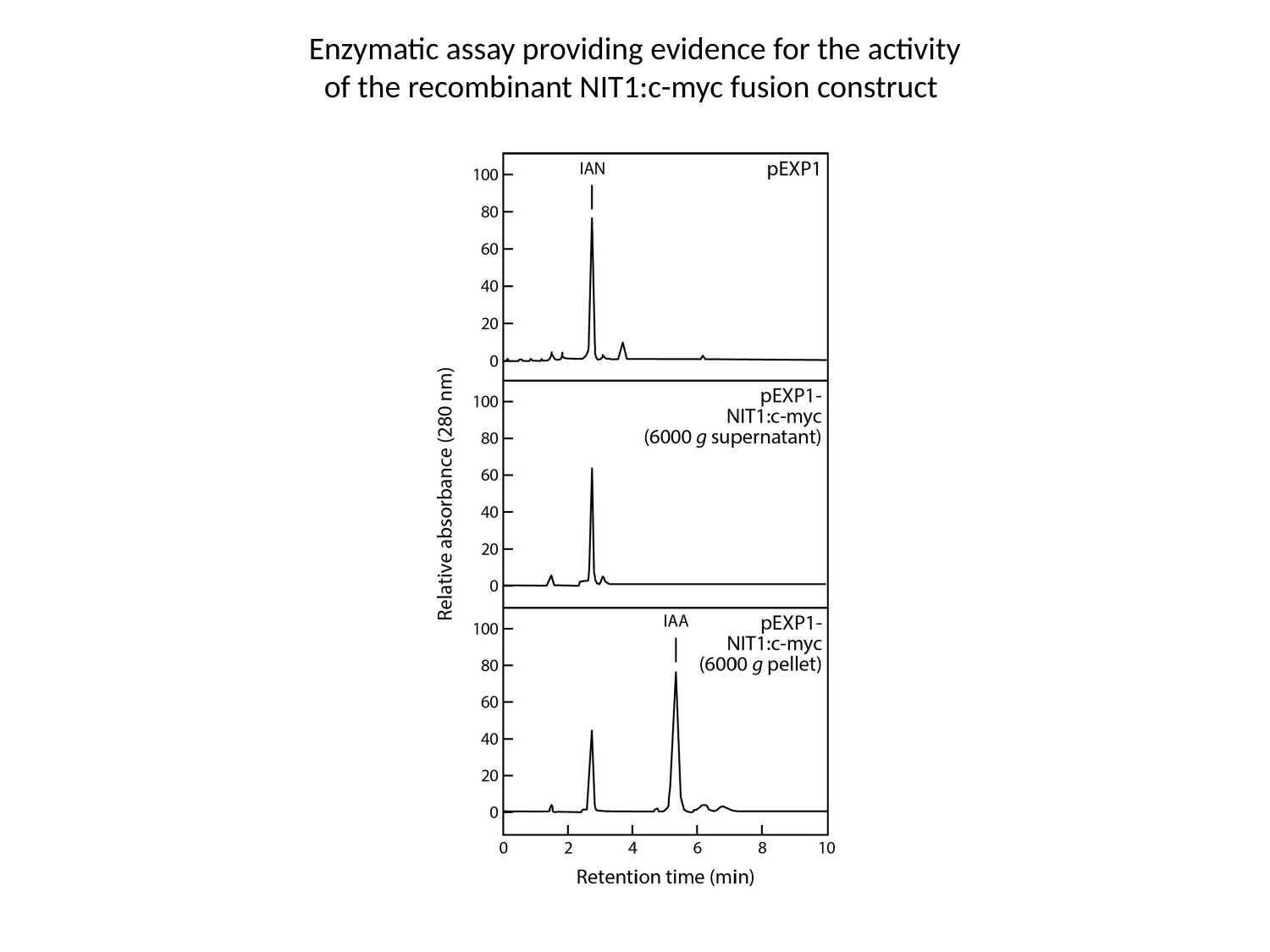

Enzymatic assay providing evidence for the activity of the recombinant NIT1:c-myc fusion construct
