# Supplementary figures and images for: Arabidopsis NITRILASE 1 Contributes to the Regulation of Root Growth and Development through Modulation of Auxin Biosynthesis in Seedlings
Source: Front Plant Sci. 2017 Jan 24;8:36. doi: 10.3389/fpls.2017.00036 (PMC5258727; doi:10.3389/fpls.2017.00036)

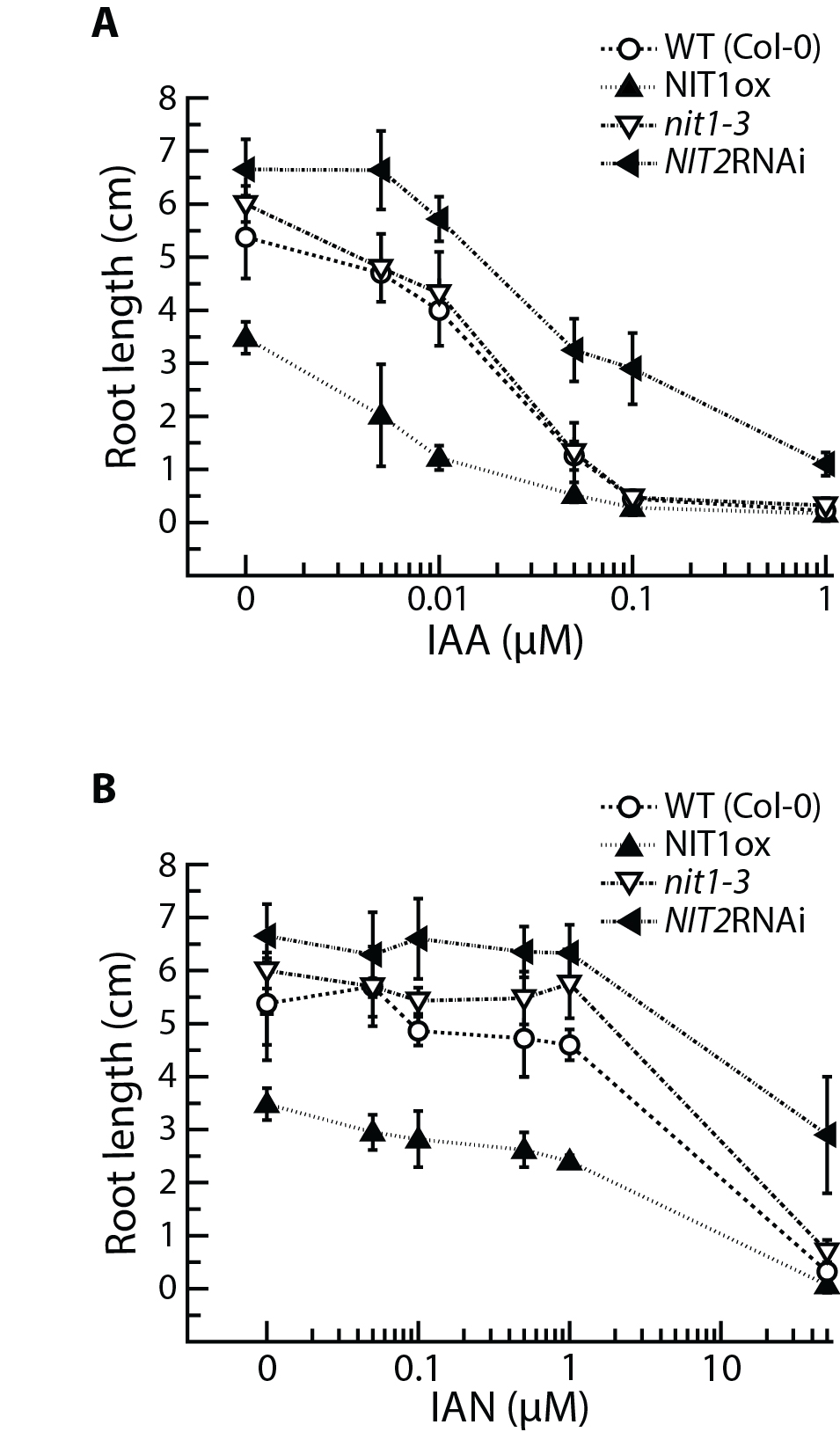

Supplement: Supplemental Image 1 — Primary root elongation of wild type, NIT1ox, nit1-3, and NIT2RNAi mutants grown on media containing either IAN or IAA. Seedlings were grown on media containing risings amounts of IAN and IAA, respectively. Since the obtained transgenic lines for NIT1ox and NIT2RNAi showed no phenotypic differences, only data obtained for NIT1ox-J5 and NIT2RNAi-8 are shown for more clarity. The numbers in the pictures indicate the concentration of IAN and IAA in the used media given in μM. For each condition and genotype at least 15 seedlings were measured. Error bars indicate the standard error of the mean. [file Image1.JPEG]
